# Pharmacists’ Mental Health during the First Two Years of the Pandemic: A Socio-Ecological Scoping Review

**DOI:** 10.3390/pharmacy11020064

**Published:** 2023-03-24

**Authors:** Liam Ishaky, Myuri Sivanthan, Mina Tadrous, Behdin Nowrouzi-Kia, Lisa McCarthy, Andrew Papadopoulos, Basem Gohar

**Affiliations:** 1 Department of Population Medicine, University of Guelph, Guelph, ON N1G 2W1, Canada; 2 Leslie Dan Faculty of Pharmacy, University of Toronto, 144 College St, Toronto, ON M5S 3M2, Canada; 3 Department of Occupational Science & Occupational Therapy, University of Toronto, 500 University Avenue, Toronto, ON M5G 1V7, Canada; 4 Centre for Research in Occupational Safety & Health, Laurentian University, 935 Ramsey Lake Rd., Sudbury, ON P3E 2C6, Canada; 5 Institute for Better Health, Trillium Health Partners, 100 Queensway West, Mississauga, ON L5B 1B8, Canada

**Keywords:** mental health, pharmacists, pandemic, antecedents, Social Ecological Model, scoping review

## Abstract

Healthcare workers have been under a great deal of stress and have been experiencing burnout throughout the COVID-19 pandemic. Among these, healthcare workers are pharmacists who have been instrumental in the fight against the pandemic. This scoping review examined the impact of the pandemic on pharmacists’ mental health and their antecedents using three databases (CINAHL, MEDLINE, and PsycINFO). Eligible studies included primary research articles that examined the mental health antecedents and outcomes among pharmacists during the first two years of the pandemic. We used the Social Ecological Model to categorize antecedents per outcome. The initial search yielded 4165 articles, and 23 met the criteria. The scoping review identified pharmacists experiencing poor mental health during the pandemic, including anxiety, burnout, depression, and job stress. In addition, several individual, interpersonal, organizational, community, and policy-level antecedents were identified. As this review revealed a general decline in pharmacists’ mental health during the pandemic, further research is required to understand the long-term impacts of the pandemic on pharmacists. Furthermore, we recommend practical mitigation strategies to improve pharmacists’ mental health, such as implementing crisis/pandemic preparedness protocols and leadership training to foster a better workplace culture.

## 1. Introduction

The COVID-19 pandemic has had a detrimental effect on healthcare workers worldwide [1]. Among these healthcare workers are pharmacists who perform a variety of roles in healthcare systems, including those related to primary, secondary, and tertiary prevention. Providing primary, secondary, and tertiary prevention strategies, pharmacists reduced transmission rates through consultations, education, administering COVID-19 vaccinations, and dispensing medications [2].

Consistent with other healthcare providers, pharmacists experienced high-stress levels during the pandemic [3]. This stress could be attributed to the rapid evolution in healthcare needs globally, combined with underlying systemic problems within the healthcare sectors, compounding the stress among healthcare workers. Recent studies conducted in various regions before the pandemic suggest that pharmacists have been experiencing poor mental health such as burnout [4,5,6,7,8,9]. For example, Durham et al. (2018) [7] found several factors associated with burnout among pharmacists, including increased non-clinical duties, inadequate time for teaching personnel, poor work environment, and feeling unappreciated. Similarly, a recent survey of community pharmacists revealed that approximately 75% of respondents were emotionally exhausted, experienced depersonalization, or felt reduced personal accomplishment [6]. Furthermore, according to Spoorthy et al. (2020) [9], several factors such as gender (women), profession (e.g., nurses), age (younger), and poor social support were associated with increased stress, anxiety, and depressive symptoms among healthcare workers during the pandemic.

With pharmacists already experiencing poor mental health before the pandemic, their stressors have likely increased considerably. However, it is unclear what type of information is available in the scientific literature concerning pharmacists’ mental health during the pandemic. While it is prudent to explore the mental health of pharmacists during the pandemic, it is equally important to statistically and qualitatively investigate factors that could influence their mental health. Identifying mental health antecedents could help inform employers and policymakers and, by extension, improve pharmacists’ mental well-being. 

Furthermore, recognizing the complexity of mental health, it is critical to conceptualize employee well-being more holistically beyond the workplace. To that end, the Social Ecological Model (SEM) can be applied to consider the intricacy between individuals’ personal, health, sociological, and environmental characteristics [10]. Initially developed by Bronfenbrenner, SEM was used to identify and understand influences on human development and has subsequently been applied in various disciplines to understand various social and health phenomena, including occupational and environmental health [11]. 

This scoping review aimed to explore the scientific literature and map information related to pharmacists’ mental health and possible antecedents using a socio-ecological lens during the first two years of the pandemic. 

## 2. Materials and Methods

### 2.1. Search Strategy

The scoping review’s protocol development, including database selection, was implemented through consultations with a scientific librarian and the research team who are well-versed in knowledge syntheses, public health (occupational health, mental health, and healthcare), and pharmacy practice. Three databases were selected for this scoping review (MEDLINE, CINAHL, and PsycINFO), given their relevance to health, mental health, and healthcare (see Appendix A). A list of search terms and subject headings was used in the initial phase of the review through word searches. Next, a comprehensive search strategy including Boolean phrases was created, which combined search terms with the subject headings and then were applied to the three databases to examine the pandemic’s impact on pharmacists’ mental health. 

Included studies focused on the mental health of pharmacists during the first two years of the pandemic. In this study, ‘mental health’ is broadly defined as one’s emotional and psychological well-being [12]. This definition encompasses diagnostic (e.g., depression) and non-diagnostic constructs (e.g., job stress). Collectively, the team decided on a broad term to collect as much detail about pharmacists’ mental well-being. Additionally, both clinical and non-clinical constructs hold considerable implications on a pharmacist’s well-being (e.g., sickness absence) and commitment to the profession (e.g., intention to leave) [13,14,15]. Furthermore, ‘antecedents’ are broadly defined as factors that could positively or negatively influence mental health. In this scoping review, an antecedent could be explained qualitatively or determined statistically. 

The inclusion criteria for this study included peer-reviewed studies published in journal articles between March 2020 (i.e., the onset of the pandemic) and May 2022 (date of search), English language only, and involved pharmacists. For studies that differentiated between healthcare groups, we synthesized the antecedents and outcomes that were relevant only to pharmacists. Quantitative, qualitative, and mixed-method studies were included to better understand pharmacists’ mental health and their antecedents. Studies where the authors pooled pharmacists with other healthcare groups without differentiation were excluded. Additionally, studies published during this period that were not conducted during the pandemic were omitted. 

### 2.2. Screening, Study Selection, and Data Charting

The software Covidence [16] was used for all screening and data extraction in this scoping review. L.I. and M.S. were the reviewers from the outset of this study. To maintain the rigor of this review, the reviewers participated in a pilot exercise. Independently, they examined the same 50 articles relevant to this review and determined whether they should be included or excluded from this review. The reviewers then discussed their findings to ensure consistency in their decision making. Initial screening was performed by examining the titles and abstracts of articles based on search criteria. All articles in the initial screening were dual-screened following the pilot screening. Inconsistencies between reviewers were discussed until a decision was reached, involving a third reviewer (B.G.). 

Two researchers conducted the charting process independently (L.I. and B.G.). For this review, we adopted an SEM for occupational and environmental health [11]. Mental health and its antecedents were examined at individual, interpersonal, organizational, community, and policy levels. The individual level includes biological and personal attitudes. The interpersonal level includes personal relationships, including family and friends. In this study, we conceptualized the community level as the relationships that pharmacists have with patients, their sense of responsibility as healthcare providers within their community, and their awareness of supports available within their community. Finally, the policy level includes government support. 

## 3. Results

**Figure 1:**
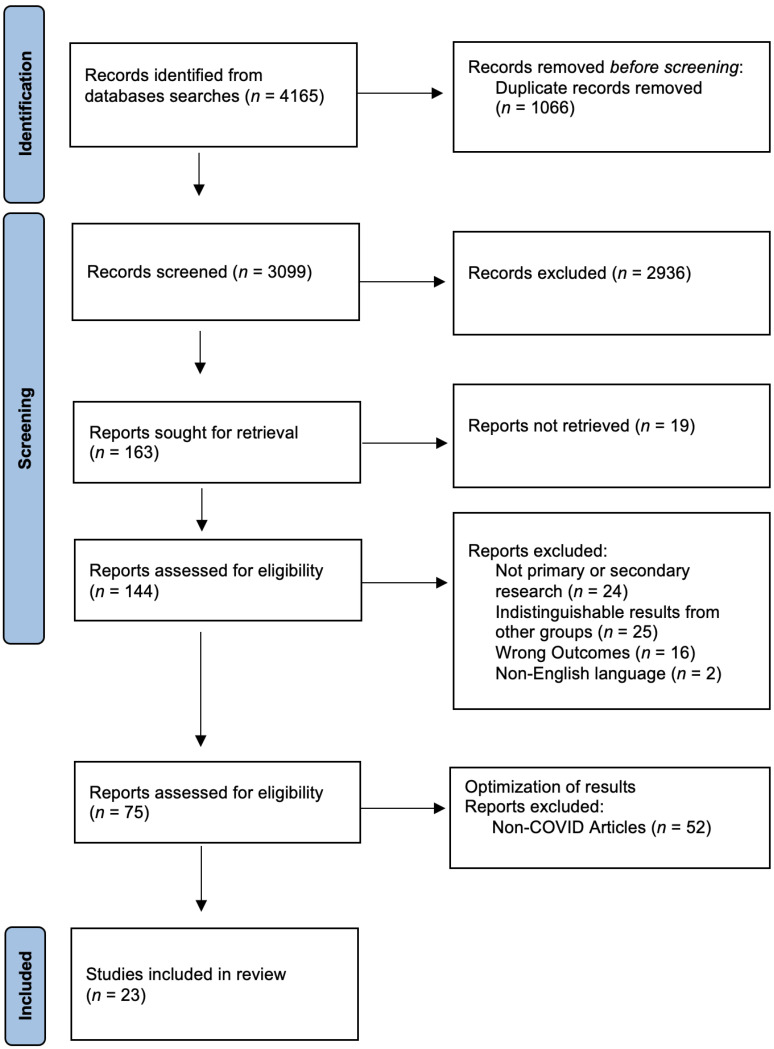
PRISMA [17,18] chart, highlighting identification, screening, and inclusion of studies.

The study’s initial search yielded 4165 articles. After title and abstract screening and removing duplicates, 75 full-text articles were considered for inclusion. Following these results, we excluded articles that did not examine the impact of COVID-19 on pharmacists’ mental health. These changes led to a total of 23 articles being used for this review. The research team followed the scoping review extension of the Preferred Reporting Items for Systematic Reviews and Meta-Analyses (PRISMA-ScR) [17] (see supplementary materials). A PRISMA chart (Figure 1) reflects these findings [18], and Table 1 provides this study’s descriptions. 

Most of the studies in this review were cross-sectional studies (*n* = 19) [3,14,15,19,20,21,22,23,24,25,26,27,28,29,30,31,32,33,34], including two that were mixed-methods [15,22]. Additionally, there was one prospective cohort study [35] and three qualitative studies [36,37,38]. Qualitative results were based on open-text analyses [36,37], interviews, and focus groups [38]. Nine studies (39%) were conducted in Asia (China [20,30], Jordan [29], Lebanon [23,27], Pakistan [21], Qatar [38], and Saudi Arabia [22]) and six studies (26%) were from European countries (France [35], Portugal [32], Serbia [25,34], Spain [31], and the United Kingdom [15]). Four studies (17.4%) were conducted in North America (Puerto Rico [37] and the United States [19,24,26]), two studies (8.7%) were from Africa (Ethiopia [28] and Nigeria [14]), and two from Australia [33,36]. Almost 75% (*n* = 17) of the studies were conducted in 2020 [15,19,20,21,22,23,24,25,26,28,30,31,33,34,35,36,37]. Two studies were conducted near the end of 2020 to early 2021 [14,23] and three were conducted in 2021 [3,27,38]. One study did not specify when the study was conducted. Approximately 70% of the studies focused on pharmacists exclusively [3,14,15,19,20,22,23,24,26,27,29,32,33,34,35,36,37,38] and the remaining examined pharmacists among other healthcare workers. Studies explored the experiences of pharmacists practicing in many care settings (e.g., community, hospital, etc.).

### 3.1. Mental Health and Antecedents

Several mental health outcomes were examined, including anxiety [3,19,20,21], burnout, depression [3,21,28], and job stress, including pandemic stressors [29,30,31,34,36,37] (Table 1). Over half of the studies (*n* = 14) examined burnout among pharmacists [14,15,19,22,23,24,25,26,27,32,33,35,36,38]. Six studies examined burnout using the Maslach Burnout Inventory [19,24,25,32,35,36], three used the Copenhagen Burnout Inventory [22,23,27], one used the Oldenburg Burnout Inventory [14], two used survey-based questions [26,34], and two explored burnout qualitatively [15,38]. Burnout severity and prevalence ranged between studies. For instance, Jones et al. (2021) reported that almost half of the sample was experiencing moderate-to-high burnout levels, with 80% having symptoms lasting up to a year [26]. They also found that nearly half who experienced moderate-to-high burnout levels also experienced secondary traumatic stress. In a Nigerian study, the authors revealed that 96% of the participants were at risk of experiencing burnout [14] In a descriptive study, Alameddine et al. (2022) [23] showed that over half of the sample experienced personal, client-based, and work-related burnout with low resiliency scores. In a cohort study, the authors determined that overall, pharmacists’ mental health was poorer during baseline compared to the follow-up [35]. The study’s results are presented at individual, interpersonal, organizational, community, and policy levels.

#### 3.1.1. Individual Level

This review revealed several factors at the individual level contributing to the mental health of pharmacists during the pandemic (Table 2). A Spanish study determined that female pharmacists reported significantly higher stress levels than male pharmacists [31]. Similarly, two studies found female pharmacists were at greater risk of burnout [14,22] while dos Santos et al. (2022) [32] found the opposite. Notably, Langran et al. (2022) [15] found that male pharmacists had slightly higher resiliency scores than their female counterparts. Meanwhile, a French study revealed no statistical relationship between gender or age with the risk of burnout [35].

Three-quarters of the studies (*n* = 3) found that younger pharmacists were at higher risk of experiencing poorer mental health, particularly burnout symptoms [14,22,27]. However, Golbach (2021) found older pharmacists were at higher risk [24]. In terms of job tenure, one study found less experienced pharmacists had greater odds of experiencing burnout [32]. Jovičić-Bata (2021) [34] found those working in chain pharmacies longer than five years were more likely to experience work stress [34].

Having a history of a mental disorder increased the risk of experiencing psychological distress, described in the study as anxiety or depression [20]. Furthermore, insufficient sleep increased the risk of burnout [27]. COVID-related factors such as having a high threat perception of contracting COVID-19, the fear of the unknown due to the pandemic, fear of working in person, or lacking the confidence in providing care to patients with COVID-19 were connected to increased burnout [15,27,32] or traumatic symptoms [31]. Finally, pharmacists whose highest degree was a bachelor’s degree and who feared making medication errors, lacked awareness of wellness programs, and had poor coping strategies or low resilience were also connected to higher risks of burnout [14,23,24,38].

#### 3.1.2. Interpersonal Level

At the interpersonal level, being single, living alone, or feeling isolated and lonely was also related to more burnout symptoms [14,32,38]. While being single or living alone was connected to burnout, Aljuffali (2022) [22] described through qualitative methods that pharmacists who are experiencing family conflicts were experiencing burnout symptoms during the pandemic. Additionally, having a dependent and finding it difficult to fulfill family responsibilities were also related to increased burnout symptoms [27,38]. Finally, Abdelsadig Mohammed (2022) [38] found that worrying about friends and family was related to burnout symptoms. Similarly, Jovičić-Bata (2021) [34] found that pharmacists who expressed low concern for their family’s health were less likely to report high work stress.

#### 3.1.3. Organizational Level

Various organizational-level factors influenced mental health. Wu et al. (2021) [30] discovered that hospital pharmacists were experiencing some level of work stress. Results from one study found that working full-time increased the odds of burnout compared to working part-time [14]. Golbach et al. (2021) [24] found that longer work hours, including longer times for completing administrative duties, were associated with greater odds of burnout. Quantitative and qualitative studies also found a connection between long work hours and experiencing burnout symptoms [22,27,32]. Likewise, Youssef et al. (2021) [27] found pharmacists who work in settings where services are provided longer than 50 h per week increased the risk of burnout. They also found that pharmacists who worked with colleagues who were COVID-19-positive were also at risk of burnout.

The pharmacists’ workload was linked to an experience of burnout symptoms [26,36,37,38]. Moreover, high workloads due to staff shortages constituted an area of concern [22,37]. In addition to higher work demands, Johnston et al. (2022) [36] discovered that reduced job resources, such as receiving less support from management and receiving inadequate training, were also contributors. Poor leadership or feeling unsupported due to a poor culture during the pandemic was also highlighted by Aljuffali et al. (2022) [22]. In contrast, Abdelsadig Mohammed et al. (2022) [38] found that burnout risk was lower in organizations where pharmacists felt a sense of autonomy and were supported by management.

Five studies examined mental health by profession [3,21,25,28,29]. In four studies, physicians served as the referent group and in one study, nurses were the referent group. In a study that examined anxiety, depression, and stress during the pandemic, Almalki et al. (2021) [21] did not find a statistical relationship. In another study, the researchers found that pharmacists had greater odds of anxiety than physicians, but no association with depression. However, GebreEyesus et al. (2021) [28] found that pharmacists had greater odds of depression than other healthcare groups. Finally, Jakovljevic et al. (2021) [25] found that pharmacists had higher scores of burnout symptoms, particularly, depersonalization than physicians and nurses, and compared to physicians, Hawari et al. (2021) [29] found that pharmacists were more likely to experience distress.

#### 3.1.4. Community Level

In a cross-sectional study, 47% reported current burnout (medium or high) and half attributed the burnout to the pandemic, with over 80% reporting their burnout lasting up to a year [33]. In a qualitative study, researchers found that the sense of duty and responsibility laid on pharmacists, and the associated worries for their organization’s profitability caused them to work beyond their limits, and thus, were more prone to experiencing burnout [38]. In a Serbian study, the authors revealed that pharmacists who reported unpleasant behaviors from clients were more likely to experience work stress [25]. On a positive note, Langran et al. (2022) [15] found in the qualitative portion of their study that despite the challenges and stressors caused by the pandemic, pharmacists noticed that, overall, clients became more understanding and were kind. Feeling unappreciated by the public was reported as a source of burnout during the pandemic, as pharmacists felt that they were supporting their patients beyond their limits [22]. In a British study, participants indicated a sense of growth and embraced technological advances, allowing them to interact with patients virtually [15]. Reportedly, participants enjoyed the efficiency and flexibility of working remotely.

#### 3.1.5. Policy Level

In an Australian qualitative study that used the demand-resource model to understand the experiences of pharmacists during the pandemic, the authors explained that unclear communication led to frustration and work stress [36]. Langran et al. (2022) [15] added that the rapid changes in practices were connected to feeling burnout symptoms. Furthermore, increased work demand was attributed to performing job duties that are usually performed by other healthcare personnel. Similarly, in another qualitative study, because physicians’ offices were closed due to policy, they felt unsupported by the government by working harder short-staffed [37]. Abdelsadig Mohammed et al. (2022) [38] found that support from the government and having established policies to deal with the pandemic were protective factors against burnout.

## 4. Discussion

This scoping review aimed to investigate pharmacists’ mental health during the first two years of the pandemic using a holistic lens. To our knowledge, this is the first knowledge synthesis that employed this framework with pharmacists within the context of the pandemic. Using the SEM [11], several mental health outcomes and their antecedents were identified among pharmacists during the first two years of the pandemic. The results of this review demonstrate that, like other healthcare professionals, pharmacists experience poor mental health, such as anxiety, burnout, and depression. Notably, a study by GebreEyesus et al. (2021) [28] revealed that pharmacists were 4.5 times more likely to experience depression during the pandemic. In contrast, being a physician served as a protective factor. Similar results were reported by Hawari et al. [29]. Finally, Jakovljevic et al. (2021) [25] found that pharmacists had higher burnout scores compared to both physicians and nurses.

At the individual level, demographic factors such as age and gender were associated with poor mental health. Out of the five studies that examined gender differences, three studies found that female pharmacists had poorer mental health than their male counterparts [22,27,31]. Additionally, one study found that male pharmacists had higher resilience scores [15]. This finding is consistent with other research that investigated gender differences among healthcare providers during the pandemic [39,40,41]. While it may be difficult to explain this association, some researchers believe it could be due to risk tolerance and cognitive appraisal of such risks. Specifically, there is evidence suggesting that men can tolerate more risk than women [40]. Notably, the relationship between gender and poorer mental health can, to some degree, be explained by the intricacy of some interpersonal factors such as having a dependent and fulfilling family responsibilities [27,38]. For example, results from an American longitudinal study revealed that women were 3.2 times more likely to exhibit depressive symptoms [42]. However, when work-family conflict was statistically accounted for in the analysis, depressive scores were decreased by 36%.

New challenges arising from the pandemic included individual, interpersonal, and organizational factors concerning the fear of contracting the virus. Specifically, some pharmacists reported fearing the “unknown” specifically during the initial phase of the pandemic. Additionally, others feared infecting their loved ones. Finally, some reported having low confidence in caring for people experiencing COVID-19, while others reported burnout when colleagues were diagnosed with COVID-19. These findings are consistent with systematic reviews that identified mental health challenges, including burnout, that impact work performance and the mental health of other healthcare workers [1,43].

Pharmacists share several common organizational-level difficulties with other healthcare workers during the COVID-19 pandemic that may have impacted their mental health. Some of these problems include increased workload, staffing shortages, increased work hours, fear of contracting COVID-19, and poor work support [27,30,44,45,46]. Pharmacists were also impacted by some challenges that were unique to their field. One challenge specific to pharmacists was that primary care offices and other medical facilities closed during the pandemic, which led to pharmacists becoming the primary care providers for a lot of patients impacted by these closures [36,38]. This role shift created an increase in workload for pharmacists and may be a contributing factor to burnout, anxiety, and depression among pharmacists during the pandemic.

At the community level, pharmacists did not feel acknowledged by the public during the pandemic, despite their important contributions [14,26]. This perceived lack of recognition most likely stems from the media attention provided to other healthcare workers such as nurses and doctors. Similarly, at the policy level, pharmacists voiced that they felt unsupported by their governments [36,37]. This feeling of a lack of support, at least in community settings, may be attributed to an absence of pharmacy integration into healthcare systems where pharmacies are viewed as healthcare retailers [47].

Similar to other retail businesses, community pharmacies require administrative and logistic support. Pharmacists are often responsible for these logistic and administrative tasks in these practice settings, which add to a pharmacist’s workload and ultimately lead to high-stress levels. However, in integrated healthcare settings such as hospitals, administrative duties are completed by others, allowing frontline workers to focus on their clinical duties. Reducing administrative demands could, in part, decrease the risk of burnout [24]. Moreover, alleviating some of the risks of burnout in pharmacists through the integration of community pharmacies into healthcare systems will in turn reduce the turnover of pharmacists, which is a burden to healthcare systems. Our review found several studies pertaining to factors that contribute to pandemic-related burnout. Since evidence suggests burnout is correlated with pharmacist turnover, understanding how to mitigate these factors is crucial in preventing further staffing shortages in pharmacies [13,48]. Without preventive steps moving forward to address burnout amongst pharmacists, there could be important implications for healthcare given the pharmacist’s role in primary, secondary, and tertiary prevention [2,13].

Most studies included in this scoping review were cross-sectional in nature or qualitative studies completed during a certain period. Thus, it is difficult to determine pharmacists’ mental health as the pandemic progresses. Interestingly, the only cohort study included in our review revealed that mental health was poorer at baseline than during follow-up, which was five months later, all in 2020 [35]. However, it is important to note that the study contained a small size (*N* = 135), and only half the participants (*n* = 67) responded to the follow-up. Nevertheless, further research investigations are warranted to examine the well-being of pharmacists as they could identify, and thus, prevent potential deleterious mental health outcomes.

Through the SEM, various antecedents that could impact pharmacists’ mental health are presented. To that end, we recommend that researchers and policymakers consider practical solutions to the identified problems. For instance, from a policy level, there should be better crisis/pandemic management strategies in place, allowing healthcare workers to act with more confidence and thus feel more supported. Strategies such as implementing protocols for handling airborne viruses, including safety procedures, would be beneficial [49]. At the organizational level, it is recommended that managerial staff consider undergoing leadership training with the aim of improving the work environment. Improved relationships among employees have favorable implications in healthcare environments, including reducing the risk of sick leave and thus improving retention [50,51].

### Limitations

As previously noted, most of the studies included were cross-sectional or qualitative in nature. Thus, it is difficult to determine pharmacists’ mental health beyond the point when data were collected. In addition, each time frame had its specific challenges throughout the course of the pandemic, which may have a varied influence on mental health. Furthermore, there was no consensus among studies on how certain constructs were defined and then measured. For instance, the authors used various questionnaires to examine burnout. However, as this was a scoping review, our aim was to map out information in the current literature. Additionally, it is important to note that a pharmacist’s scope of practice varies from region to region, and therefore demands may differ. Moreover, the impacts of government policy surrounding COVID-19 were likely different between countries, which could have influenced pharmacy practice, and by extension, the pharmacist’s well-being.

## 5. Conclusions

Pharmacists are instrumental professionals that provide primary, secondary, and tertiary preventive care. During the pandemic, this was apparent as pharmacists were active frontline healthcare workers that remained accessible to the public when other healthcare workers were unavailable. Using the SEM, several mental health antecedents at the individual, interpersonal, organizational, community, and policy levels were identified. In general, the results revealed that pharmacists experienced high levels of burnout, anxiety, depression, and stress throughout the pandemic. These challenges are detrimental to pharmacists and the healthcare system. Further research is needed to examine pharmacist mental health in relation to the ongoing pandemic and post-pandemic environment to prevent future staff turnover and damage to our healthcare system. In addition to the exploration of further integrating pharmacists within the healthcare system, we recommend that managerial staff undergo leadership training to improve the workplace culture, and, at the policy level, we encourage more efforts related to pandemic preparedness.

## Figures and Tables

**Table 1:** Study Descriptions.

First Author, Year/Origin	Origin	Study Design	Pandemic Period	Pharmacist Setting and Sample Size	Mental Health Outcome(s) Specific to Pharmacists
Almalki, 2021 [3]	Saudi Arabia	Cross-Sectional	January–March 2021	Community and hospital; (*N* = 501)	Anxiety; depression; stress
Hedima, 2022 [14]	Nigeria	Cross-sectional	December 2020–February 2021	Community, hospital, academic, pharmaceutical industry, primary care; (*N* = 426)	Burnout
Langran, 2022 [15]	United Kingdom	Mixed-method (Cross-sectional)	June–July 2020	Community; hospital; general practice; (*N* = 199)	Burnout; resilience; well-being
Bakken, 2021 [19]	United States	Cross-Sectional	August–September 2020	Community hospital and health systems; (*N* = 439)	Burnout; emotional health (including anxiety and depression)
Yang, 2021 [20]	China	Cross-sectional	February 2020	Hospital; (*N* = 365)	Psychological distress, including anxiety, depression
Hayat, 2021 [21]	Pakistan	Cross-sectional	May–June 2020	General practice (among other groups; pharmacist *n* = 75)	Anxiety; depression
Aljuffali, 2022 [22]	Saudi Arabia	Mixed-Method (Cross-sectional)	June–September 2020	Community and hospital; (*N* = 502)	Burnout
Alameddine, 2022 [23]	Lebanon	Cross-sectional	December 2020–January 2021	Community; (*N* = 459)	Burnout/resilience
Golbach, 2021 [24]	United States	Cross-sectional	October–November 2020	Hematological oncology; (*N* = 550)	Burnout
Jakovljevic, 2021 [25]	Serbia	Cross-sectional	June–December 2020	General practice (among other groups; pharmacist *n* = 40)	Burnout
Jones, 2021 [26]	United States	Cross-sectional	April–May 2020	Unspecified; (*N* = 484)	Burnout; secondary trauma stress
Youssef, 2021 [27]	Lebanon	Cross-sectional	February–March 2021	Community; (*N* = 387)	Burnout
GebreEyesus, 2021 [28]	Ethiopia	Cross-sectional	November 2020	Unspecified; (among other groups; pharmacist *n* = 38)	Depression
Hawari, 2021 [29]	Jordan	Cross-sectional	April–May 2020	Community and hospital; *(n* = 166)	Stress
Wu, 2021 [30]	China	Cross-sectional	August–September 2020	Hospital (among other groups; pharmacist *n* = 249)	Job Stress
Baldonedo-Mosteiro, 2022 [31]	Spain	Cross-sectional	April 2020	Community (among other groups; pharmacist *n* = 739)	Stress
dos Santos, 2022 [32]	Portugal	Cross-sectional	Unknown	Community; hospital; (*N* = 1362)	Burnout
Johnston, 2021 [33]	Australia	Cross-sectional	April–June 2020	Community; hospital; (*N* = 647)	Burnout; survey-based questions
Jovičić-Bata et al., 2021 [34]	Serbia	Cross-sectional	April–May 2020	Community (chains and independent); (*N* = 392)	Stress; coping with burden
Lange, 2022 [35]	France	Cohort	Initial: April 2020; follow-up: September 2020	Community (initial *n* = 135; follow-up *n* = 67)	Burnout; perceived stress; post-traumatic stress
Johnston, 2022 [36]	Australia	Qualitative (open text)	April–June 2020	Community; hospital; (*N* = 215)	Stressors during the pandemic
Silva-Suarez, 2022 [37]	Puerto Rico	Qualitative (Open-text analysis)	May–June 2020	Community; (*N* = 233)	Stressors during the pandemic
Abdelsadig Mohammed, 2022 [38]	Qatar	Qualitative (focus groups and interviews)	February–April 2021	Community; (*N* = 45)	Burnout

Note: “*N*” indicates that pharmacists represented the entire study’s sample size and “*n*” outlines the sample size for cohort studies or if pharmacists were a subsample of the entire study’s sample size.

**Table 2:** Mental health antecedents using the Social Ecological Model.

Levels	Mental Health	Antecedents
Individual: Risk	Psychological Distress (including anxiety and depression)	History of a mental illness [20]
	Burnout/resilience	Low resiliency [23]; younger pharmacists [14,15,22,27]; older pharmacists [24]; women [14,15,22,27,31] but men had higher depersonalization [25,33]; less work experience [32]; bachelor’s degree (vs. graduate) [14]; insufficient sleep [27]; lacking confidence [32]; high COVID-19 threat perception [27]
	Job Stress	Feeling scared or unsafe [15,36,37] * [31]
Individual: Protective	Burnout	Coping skills [38] *
Interpersonal: Risk	Burnout	Single [14,23]; family/marital issues [22] *; having dependents [27]; fulfilling family role [38] *; isolated/lonely [38] * [32]; fear of infecting family members [37,38] *
Interpersonal: Protective	Burnout	Working from home [15] *; social support [15,38] *
	Stress	Low safety concern for family [34]
Organizational: Risk	Burnout	High workload [26,37,38] * [19,26]; increased work hours [22,27]; difficulty connecting with colleagues/poor work culture [22] * [19]; colleague diagnosed with COVID-19 [27]; staff shortage [22,37,38] *; full-time (vs. part-time) [14]; vs. other professions [25]; Community vs. independently owned [34]; unrealistic work expectations [15] *; poor leadership [15] *; inability to reach physicians due to lockdown [38]
	Psychological Distress (including anxiety and depression)	Poor working conditions [20]
	Anxiety	vs. physicians [21]
	Job stress	High report of job stress [30]; vs. other professions [29,34]; high workload; supporting others in their role *; completing duties of other roles [36] *; poor leadership [36] *; inadequate training [36] *
	Depression	vs. other occupations [28]
Organizational: Protective	Burnout	Peer support [15,38] *; opportunities for professional development [15] *
Community: Risk	Burnout	Difficulty connecting with patients [19]; lack of awareness of wellness programs; sense of responsibility to community;
	Job Stress	Inability to reach physicians due to lockdown [37] *; feeling unappreciated/negative patients [36] * [34]
Community: Protective	Burnout	Technological advances allowed for more patient interactions [15] *; positive shift in patient attitude [15] *
Policy: Risk	Stress	Feeling unsupported by government [37] *; receiving unclear communication [36] *
Policy: Protective	Burnout	Supported by the government [38] *

* Qualitative descriptions (i.e., non-statistical) Note: Two studies [3,35] are not presented, as the identified antecedents did not influence mental health.

## Data Availability

To receive selection results, please contact the corresponding author.

## Supplementary Materials

The following supporting information can be downloaded at: https://www.mdpi.com/article/10.3390/pharmacy11020064/s1, Preferred Reporting Items for Systematic reviews and Meta-Analyses extension for Scoping Reviews (PRISMA-ScR) Checklist.

## Appendix A

Search Strategy/Search Example:

PsycINFO

({Pharmacists} OR Pharmacist*) AND ({Burnout} OR Burnout) AND ({Occupational Stress} OR {Compassion Fatigue} OR Occupational Stress) AND ({Psychological Stress} OR Stress) AND ({Major Depression} OR Depressive Disorder*) AND ({Anxiety Disorders} OR Anxiety Disorder*) AND (Health Personal Alert Fatigue) AND ({Tranquilizing Drugs} or Anti-Anxiety Agent*) AND ([Antidepressant Drugs} OR Antidepressive Agent*) AND ({Cognitive Behavior Therapy} OR {Acceptance and Commitment Therapy} OR {Cognitive Processing Therapy} OR {Prolonged Exposure Therapy} OR Cognitive Behavioral Therapy) AND ({Mental Disorders} OR Mental Disorder*) AND ({Mental Health} OR Mental Health) AND ({Post-Traumatic Stress Disorder} OR Post-Traumatic Stress Disorder) AND ({Emotional Exhaustion} OR Mental Fatigue) AND ({Emotions} OR {Contempt} OR {Desire} OR {Emotional Content} OR {Emotional Disturbances} OR {Emotional Health} OR {Emotional Processing} OR {Emotional Regulation} OR {Emotional States} OR {Emotional Style} OR {Emotional Support} OR {Expressed Emotion} OR {Negative Emotions} OR {Positive Emotions} or Emotion*).
